# Potential Factors of Corneal Endothelial Cells for Progression in Children with Uveitis

**DOI:** 10.1155/2021/8432774

**Published:** 2021-12-28

**Authors:** Xu Chen, Yi Shao, Shi-Nan Wu, Shan-Bi Zhou

**Affiliations:** ^1^Department of Ophthalmology, Haidian Section of Peking University Third Hospital (Beijing Haitian Hospital), Beijing 100080, China; ^2^Department of Ophthalmology, The First Affiliated Hospital of Nanchang University, Nanchang, 330006 Jiangxi Province, China; ^3^Department of Ophthalmology, The University-Town Hospital of Chongqing Medical University, Chongqing 401331, China

## Abstract

**Objective:**

To observe the morphological changes and abnormal structure of corneal endothelial cells in children with uveitis, to analyze the related factors affecting the morphological changes of corneal endothelial cells, and to explore the clinical application of a corneal endothelial microscope in children with uveitis.

**Methods:**

The corneal endothelial cells of 70 patients with uveitis were photographed with the Topcon SP-3000 noncontact corneal endothelial microscope, and the corneal endothelial cell density (CD), average cell area (AVE), coefficient of variation of the cell area (CV), and percentage of hexagonal cells (PHC) were measured with the IMAGEnet system. Twenty-eight patients (56 eyes) with monocular uveitis were selected, with the affected eyes (28 eyes) as the experimental group and the contralateral healthy eyes (28 eyes) as the control group. The corneal endothelial cell parameters between the two groups were statistically analyzed. The parameters of corneal endothelial cells in 70 children with uveitis were compared, and the effects of the course of the disease, inflammatory cells in the anterior chamber, and posterior corneal deposition (KP) on the parameters of corneal endothelial cells were analyzed.

**Results:**

There are four abnormal forms of the corneal endothelium in children with uveitis: enlarged cell area gap, irregular cell shape, blurred intercellular space, and cell loss. KP showed irregular high reflective white spots in the corneal endothelial microscope images, surrounded by dark areas, and existed in all the eyes with dusty KP found in slit lamp examination and a small number of eyes without obvious KP. Comparing the corneal endothelial cell parameters between the experimental group and the control group, it was found that the corneal endothelial CD and PHC of the former were lower than those of the latter, and the difference was statistically significant (*P* < 0.001 and *P* = 0.018, respectively). The AVE and CA of the former were higher than those of the latter (*P* = 0.013 and *P* = 0.046, respectively). The corneal endothelial cell density of the eyes with a course of the disease of more than 1 year was lower than that of the eyes with a course of the disease less than 1 year, the coefficient of variation of the corneal endothelial cell area of the eyes with KP was higher than that of the eyes without KP, and the difference was statistically significant (*P* = 0.003 and *P* = 0.030, respectively).

**Conclusion:**

Corneal endothelial microscopy is one of the important methods for the detection of uveitis with high sensitivity. The change of morphological parameters of corneal endothelial cells is one of the important indexes to assist in the diagnosis of uveitis and can be further promoted in ophthalmological examination.

## 1. Introduction

Uveitis in children refers to uveitis in people under the age of 16 (or 15) years [1]. The incidence of uveitis is usually lower in this population than in young adults. Almost all types of uveitis that occur in adults also occur in children, but there are also some types that are specific to the latter; as many of these have no obvious irritating symptoms, chronic uveitis in children is not easily detected at early stages of the disease. Uveitis in children is mostly associated with chronic inflammation, and in general, the visual prognosis of patients is worse than that of adults with complications that include cataract, banded corneal degeneration, secondary glaucoma, and strabismus amblyopia [2]. Additionally, long-term intraocular inflammation can damage corneal endothelial cells that are in direct contact with aqueous humor [3].

A corneal endothelial microscope is used to determine whether corneal endothelial cells in children with uveitis are damaged and identify the factors causing morphologic changes in the cells. The Topcon SP-3000 corneal endothelial microscope (Topcon, Tokyo, Japan) is a new type of noncontact corneal endothelial microscope that can collect corneal endothelial images in vivo for analyses of corneal endothelial cell size, shape, and structural abnormalities; combined with the IMAGEnet system, corneal endothelial cell density (CD), average cell area (AVE), coefficient of variation of the cell area (CV), and percentage of hexagonal cells (PHC) are automatically calculated. Qualitative and quantitative data can be used to assess the functional state of the corneal endothelium, which can aid in the diagnosis of ophthalmopathies.

In this study, the corneal endothelium of 70 cases (140 eyes) with uveitis was qualitatively and quantitatively examined using a Topcon SP-3000 corneal endothelial microscope and IMAGEnet system. The corneal endothelial cell parameters of affected eyes (28 eyes) and contralateral healthy eyes (28 eyes) of 28 monocular children with uveitis were compared. We also compared the parameters between sexes and between left and right eyes and analyzed the effects of the disease course, inflammatory cell infiltration into the anterior chamber, and posterior corneal keratic precipitate (KP) deposition. The aim of the study was to evaluate morphologic changes in corneal endothelial cells and the influencing factors and explore the clinical application of the corneal endothelial microscope in children with uveitis.

## 2. Methods

### 2.1. Study Subjects

From November 2009 to March 2010, a total of 70 patients (140 eyes) with uveitis were examined including 28 patients with monocular uveitis (28 eyes) and their contralateral healthy eyes (28 eyes) and 42 cases (84 eyes) with binocular uveitis. There were 32 males and 38 females, with an average age of 11.77 ± 4.08 years (range: 4–29 years). The best-corrected visual acuity (BCVA) was HM/20 cm~1.5, and the shortest and longest disease course was 1 month and 15 years, respectively. Inclusion criteria for children with monocular uveitis were as follows: (1) age of the first onset < 16 years; (2) uveitis diagnosed for a single eye; (3) BCVA of contralateral healthy eye ≥ 1.0, with no organic lesions; and (4) normal intraocular pressure and anterior segment and fundus examinations of the contralateral healthy eyes. Inclusion criteria for patients with binocular uveitis were as follows: (1) age of the first onset < 16 years and (2) uveitis diagnosed for both eyes. Exclusion criteria for all patients were as follows: (1) corneal contact lenses, (2) hypertension, (3) diabetes, (4) internal eye surgery or treatment, (5) eye trauma, or (6) other eye diseases not secondary to uveitis.

This study was a retrospective study. All procedures in this study were in compliance with the Helsinki Declaration. The Medical Ethics Committee of the First Affiliated Hospital of Nanchang University approved the study (no. 2018021). All participants and their guardians were fully aware of the purpose, content, and potential risks of this study and signed an informed consent form prior to enrollment.

#### 2.1.1. Instruments and Inspection Steps

*(1) Eye Examination and Methods*.

1. Distant vision: the international standard *E* visual acuity chart was used to measure the vision at a distance of 5 m
2. Best-corrected visual acuity examination: all subjects used subjective refractive examination to obtain the best-corrected visual acuity according to the naked visual acuity of the subjects
3. Anterior segment examination: all subjects used a slit lamp to examine the cornea, sclera, anterior chamber, iris, pupil, and lens
4. Fundus examination: all subjects used a direct ophthalmoscope to examine the vitreous body and fundus and fundus fluorescein angiography or/and indocyanine green angiography if necessary

#### 2.1.2. Microscopic Examination of the Corneal Endothelium

*(1) Equipment*. A noncontact corneal endothelial microscope (Topcon SP-3000 Topcon Optical Co., Ltd., Japan; photography range 0.25 × 0.5 mm; magnification 150x; thickness measurement 0.01 mm accuracy) was used for corneal endothelial cell photography. The corresponding computer-aided measurement and analysis system (IMAGEnet) was used to process and analyze the images. The parameters include CD, minimum cell area, maximum cell area, AVE, the standard deviation of the cell area, CV, and PHC.

#### 2.1.3. Examination Methods and Steps

(1) The subject's head was positioned on the bracket. (2) The central measurement area was selected, and the image was taken automatically. The central corneal endothelial cells were photographed three times, and the best frame was selected for preservation. (3) The images were assessed using direct observation and qualitative analysis. (4) Corneal endothelial cells were quantitatively analyzed by the IMAGEnet system. (5) The results were printed after analysis

#### 2.1.4. Image Processing and Analysis of Corneal Endothelial Cells

*(1) Qualitative and Quantitative Analysis*. The qualitative analysis of corneal endothelial cells included consistency of cell size and morphology and identification of any intracellular or intercellular abnormality in structure. The quantitative analysis of corneal endothelial cells in this study included CD, AVE, CV, and PHC.

### 2.2. Statistical Analysis

Data were analyzed using SPSS v26 software (SPSS Inc., Chicago, IL, USA); the significance level for all statistical analyses was *P* < 0.05. Corneal endothelial cell parameters were compared between the experimental and control groups with the paired-sample *t*-test and between the 2 sexes and left and right eyes with the independent sample *t*-test. The effects of the disease course, inflammatory cell infiltration into the anterior chamber, and KP accumulation on corneal endothelial cell parameters were analyzed by factorial analysis of variance (ANOVA). The Pearson correlation analysis was performed to analyze the correlations among CD, AVE, CV, and PHC, and linear regression analysis was carried out using Prism v8.0 software (GraphPad, La Jolla, CA, USA).

## 3. Results

### 3.1. Patients with Monocular Uveitis

A total of 28 children with monocular uveitis met the inclusion criteria. There were 19 males (19 eyes) and 9 females (9 eyes) with a mean age (±SD) of 11.57 ± 3.66 years (range: 5–19 years). The BCVA was HM/20 cm~1.2, and the shortest and longest disease course was 1.5 months and 5 years, respectively. The control group consisted of the contralateral healthy eyes of patients with monocular uveitis; the BCVA was ≥1.0, and there were no abnormalities in the anterior segment and fundus examinations.

### 3.2. Eyes with Uveitis

Among the 70 children (112 eyes) with uveitis, there were 32 males (46 eyes), 38 females (66 eyes), 56 left eyes, and 56 right eyes (Table 1). The patients were divided into the following groups according to the disease course, presence of inflammatory cells in the anterior chamber, and presence of KP. The short and long disease course groups comprised 22 patients (35 eyes) who had had uveitis for ≤1 year and 48 patients (77 eyes) with uveitis for >1 year, respectively. There were 64 eyes with and 48 without inflammatory cells in the anterior chamber by slit lamp examination and 69 eyes with uveitis without KP and 43 with KP in the slit lamp and corneal endothelial microscopy examinations.

### 3.3. Qualitative Analysis of Corneal Endothelial Cells

**Figure 1:**
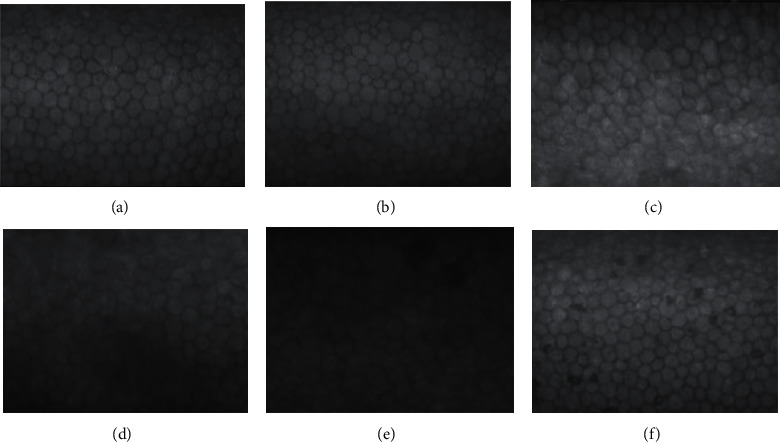
Qualitative analysis of corneal endothelial cells: (a) corneal endothelium of the normal eye in a patient with unilateral juvenile and pediatric uveitis; (b) changes in cell size uniformity of the corneal endothelium in juvenile and pediatric uveitis eyes; (c) changes in cell morphology consistent with the corneal endothelium in juvenile and pediatric uveitis eyes; (d) blur intercellular space of the corneal endothelium in juvenile and pediatric uveitis eyes; (e) cell loss of the corneal endothelium in juvenile and pediatric uveitis eyes; (f) KP in juvenile and pediatric uveitis eyes.

In the 28 healthy eyes, the corneal endothelial cells were similar in size and arranged in an orderly fashion; most of the cells had a typical hexagonal structure with moderate reflection and an intercellular space showing a dark reflection and no abnormal structures such as vacuoles or dropwise warts. A slightly darker reflection was seen in the center of some cells. The average density of endothelial cells was 3182.8 ± 253.7 cells/mm^2^ (range: 2638.7 to 3534.3 cells/mm^2^) (Figure 1(a)). In contrast, in patients with uveitis, larger corneal endothelial cells were observed that were usually adjacent to missing cells and appeared to have expanded to fill the gap; meanwhile, other cells were smaller as they were squeezed between cells, with the result that the cell area was increased in some cases and decreased in others, yielding an uneven and disordered corneal endothelium (Figure 1(b)). A higher proportion of corneal endothelial cells in eyes with uveitis had an irregular shape compared to the cells in healthy eyes: the hexagonal structure was no longer visible and the cells were triangular, square, pentagonal, or even round (Figure 1(c)). The dark reflection of the intercellular space was incomplete, blurred, or disordered, and the shape of the cells was indistinct or else the cells were fused into slices or dark areas (Figure 1(d)). Single or multiple corneal endothelial cells were missing, appearing as a black area, while the surrounding cells were structurally intact (Figure 1(e)). In the slit lamp examinations, 24 eyes had KP deposits that appeared as scattered, irregular, highly reflective white spots surrounded by dark areas; these were mostly <1 corneal endothelial cell diameter and located inside, between, or across cells (Figure 1(f)). These abnormal spots were also present in 17 eyes with no obvious KP deposition by slit lamp examination but not in 2 eyes with lanolin KP deposits.

### 3.4. Quantitative Analysis of Corneal Endothelial Cells

The cell density and the percentage of hexagonal of corneal endothelial cells in patients with uveitis were lower than those in healthy controls (*P* < 0.001 and *P* = 0.018, respectively). The average cell area and coefficient of variation in patients with uveitis were significantly higher than those in healthy subjects (*P* = 0.013 and *P* = 0.046, respectively). More details are shown in Table 2.

### 3.5. Comparison of Corneal Endothelial Cell Parameters between Sexes and Eyes

There were no significant differences in any of the corneal endothelial cell parameters between male and female children with uveitis (*P* < 0.05; Table 3), nor between the right and left eyes with uveitis (*P* > 0.05; Table 4).

### 3.6. Analysis of Factors Affecting Corneal Endothelial Cell Parameters

In order to clarify the effects of the disease course, inflammatory cell infiltration into the anterior chamber, and KP deposition on corneal endothelial cell parameters in children with uveitis, we analyzed 112 eyes by factorial ANOVA (Table 5). The main effects of the disease course on corneal endothelial cell density (*F* = 8.987, *P* = 0.003) and of KP on the coefficient of variation of the cell area (*F* = 4.842, *P* = 0.030) were statistically significant, and the interaction of the disease course, anterior chamber inflammatory cell infiltration, and KP deposition had a statistically significant effect on the density of corneal endothelial cells (*F* = 6.224, *P* = 0.014). The main effects or interactions of other factors had no significant effects on other parameters of corneal endothelial cells (*P* > 0.05; Table 6).

**Figure 2:**
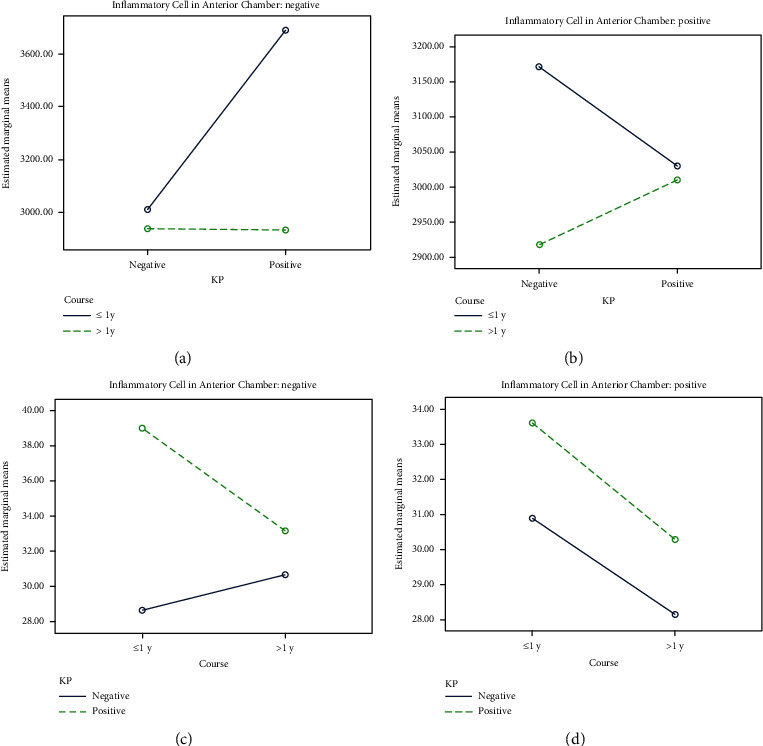
Mean corneal endothelial cell density and mean corneal endothelial cell area coefficient of variation: (a) inflammatory cell in the anterior chamber is negative; (b) inflammatory cell in the anterior chamber is positive; (c) inflammatory cell in the anterior chamber is negative; (d) inflammatory cell in the anterior chamber is positive.

**Figure 3:**
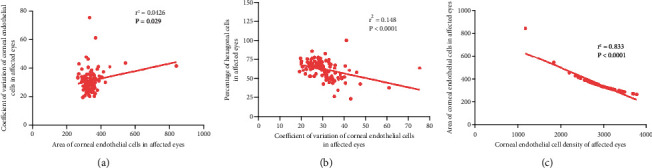
Correlation of corneal endothelial cell parameters in children with uveitis.

The density of corneal endothelial cells in uveitis eyes with a disease course > 1 year was lower than that in diseased eyes with a course < 1 year (Figures 2(a) and 2(b)). The coefficient of variation of the corneal endothelial cell area in affected eyes with KP was higher than that in affected eyes without KP (Figures 2(c) and 2(d)). The coefficient of variation of the cell area in uveitis eyes was positively correlated with the cell area (*r*^2^ = 0.0426; *P* = 0.029). There was a negative correlation between the percentage of hexagonal cells and coefficient of variation (*r*^2^ = 0.148; *P* < 0.0001). The corneal endothelial cell area was negatively correlated with corneal endothelial cell density (*r*^2^ = 0.833; *P* < 0.0001) (Figure 3).

## 4. Discussion

Uveitis in children accounts for 1.2%–13.2% of total uveitis cases. Although it often causes banded corneal degeneration, vision is affected only when there is transverse zonal corneal degeneration. Banded degeneration of the cornea is mainly caused by chronic anterior uveitis involving the deposition of calcium in the corneal epithelial basement membrane and Bowman membrane; long-term intraocular inflammation may also damage corneal endothelial cells. The effect of uveitis on corneal endothelial cells has not been widely studied, and the mechanism of KP deposition is not well understood, in part due to the technical difficulty of corneal endothelium sampling [4].

As the innermost layer of the cornea, the endodermis is composed of a monolayer of endothelial cells attached to the posterior elastic layer and contacting the anterior aqueous humor. The cells have a Na^+^-K^+^ATP enzyme pump function [5] and constantly drain water molecules from the stroma into the anterior chamber, thereby maintaining a state of dehydration in the former and keeping the cornea transparent. Endothelial cells cannot be regenerated [6]. When the human corneal endothelium is damaged, healing occurs through the expansion of cells and their formation of a continuous layer on the inner surface of the cornea [7]. For example, following the loss of corneal endothelial cells due to pathologic changes, there is an increase in the area of single cells and a decrease in cell density; the injured area is covered with adjacent cells that expand and migrate into the injury site. Damage to endothelial cells that exceeds a certain limit can lead to corneal edema and thickening and even vision loss or blindness [8].

So far, the corneal endothelium can be observed by different instruments in clinical ophthalmology. The slit lamp technique using high magnification and specular reflection alone cannot be used to evaluate the size and number of corneal cells. Therefore, we need to rely on other diagnostic techniques, such as noncontact corneal endothelial cell microscopy [9]. A noncontact corneal endothelial microscope is commonly used to evaluate corneal endothelial cells in vivo. The measurement data obtained using the microscope have good repeatability and stability. It is recommended that corneal endothelial cells should be analyzed from the clearest image combined with the Topcon IMAGEnet system [10]. For the same set of images captured by the same examiner, the IMAGEnet system allows reliable quantification of corneal endothelial cell density and average cell area and can be used to dynamically assess changes in cell density, morphology, and function caused by pathologic stimuli such as drug toxicity and inflammation.

### 4.1. Corneal Endothelial Cells of Normal Children

The typical morphology of corneal endothelial cells in normal children is hexagonal; the cells have a clear boundary and are regularly arranged in a straight line and are tightly packed and well-defined. This was observed in the present study in images of corneal endothelial cells in healthy eyes of children with monocular uveitis. In adults with uveitis, corneal endothelial cells show increased atypia and are loose with indistinct boundaries; the corneal surface loses its smooth boundary and becomes irregular, the intercellular space increases, and there are dark areas formed by local endothelial cell apoptosis and loss. The density of corneal endothelial cells and the percentage of hexagonal cells in the central area of the cornea are significantly higher while the cell area is smaller in children than in adults. With increasing age, the density of corneal endothelial cells and percentage of hexagonal cells decrease, and the cell area and the coefficient of variation increase [11]. Therefore, we believe that it is more reliable to examine the damage to corneal endothelial cells caused by uveitis in children rather than in adults, as the influence of aging-related changes in corneal endothelial morphology is eliminated.

### 4.2. Changes in the Corneal Endothelium of Children with Uveitis

By microscopic examination of the corneal endothelium, we observed 4 major differences in children with uveitis as compared to the normal control group—namely, a larger cell area, irregular cell shape, blurred intercellular space, and cell loss. In addition, in all eyes with KP deposition and the small number of eyes in which this was not detected, the KP appeared as irregular highly reflective white spots surrounded by dark areas, which were not observed by slit lamp examination. In the eyes of uveitis patients with KP, most showed dusty KP, but there was no obvious KP or dark areas in the corneal endothelium of eyes with lanolin KP regression. This may be because the amount of lanolin KP in the affected eyes was too low and did not represent typical cases of regression. KP is deposited in the central area of the cornea, which is difficult to photograph. At the same time, the low magnification power of the noncontact corneal endothelial microscope limits the detection and detailed examination of structural abnormalities.

A previous study using a contact corneal endothelial microscope to prospectively observe 13 patients with monocular acute anterior uveitis found that newly formed KP appeared as a dense white reflection, with a size ranging from about 1–2 to 5–10 corneal endothelial cells in diameter [12]. With the improvement of uveitis, the morphology of KP changed markedly: the old KP appears as a white reflection in the center of cells surrounded by large shadows that occasionally replaced the original KP with a shadow or empty area. Corneal endothelial vacuoles in shaded or defective areas are observed in patients with recurrent uveitis. The dusty KP in our patients was also scattered, but the size was mostly <1 corneal endothelial cell diameter. The loss of corneal endothelial cells that we observed may be attributable to corneal endothelial vacuoles or a dark area left after the original KP disappeared as the disease improved; this was accompanied by a gradual restoration of the boundary between adjacent cells. Thus, the disease has entered the recovery stage when the dark area of the endothelium in acute iridocyclitis appears discontinuous, which cannot be observed by slit lamp examination. Our results demonstrate that corneal endothelial microscopy has greater clinical utility than slit lamp and other examinations for the diagnosis and monitoring of uveitis.

The density of corneal endothelial cells in 28 children with uveitis was 2843.1 ± 465.7/mm^2^, and the percentage of hexagonal cells was 59.8% ± 14.5%; these values were significantly lower than in contralateral healthy eyes, in which the density was 3182.8 ± 253.7/mm^2^ and percentage of hexagonal cells was 66.9% ± 12.5%. Meanwhile, the average cell area and coefficient of variation of the corneal endothelium in uveitis eyes were 367.6 ± 105.9 *μ*m^2^ and 32.1% ± 6.4%, respectively, which was significantly higher than in contralateral healthy eyes (316.2 ± 26.0 *μ*m^2^ and 28.6% ± 5.2%, respectively). We found no significant differences in corneal endothelial cell parameters between the 2 sexes and between left and right eyes in children with uveitis, indicating that the effects of uveitis on corneal endothelial cells in children are not influenced by sex or laterality.

The mechanism underlying abnormalities in corneal endothelial cells in the eyes of children with uveitis remains to be elucidated. Tumor necrosis factor (TNF)-*α* is involved in the occurrence and development of uveitis [13]; it is known to induce corneal endothelial cell injury by destroying the integrity of the corneal endothelial barrier, fracturing the peripheral junction actin ring, rearranging the apical junction protein body, and promoting the disintegration of microtubules. These effects are mediated by transient activation of p38 mitogen-activated protein kinase (MAPK) and effects are abrogated by inhibitors of p38 MAPK and downstream factors affecting the cytoskeleton [14]. Agents that enhance microtubule stabilization can also mitigate the destruction of the corneal endothelial cell barrier caused by TNF-*α*-induced inflammation in transplant rejection and uveitis [15].

### 4.3. Factors Affecting the Changes of Corneal Endothelial Cell Parameters

In this study, we analyzed the effects of the course of the disease, anterior chamber inflammatory cells, and KP on the parameters of corneal endothelial cells, because these three factors may be interrelated and restricted; that is, there may be interaction. Therefore, the analysis of variance of factorial design can not only test whether the differences between the factors and levels are statistically significant, but also test the interaction between the factors at the same time. From the analysis results, it can be seen that the main effect of KP on the coefficient of variation of cell area is statistically significant, and the main effect of the course of disease and the interaction of course, anterior chamber inflammatory cells and KP on corneal endothelial cell density are statistically significant.

Combined with the average coefficient of variation of corneal endothelial cell area, regardless of the course of disease and the presence of inflammatory cells in the anterior chamber, the coefficient of variation of corneal endothelial cell area in eyes with KP was higher than that without KP. A United Kingdom-based study by Pillai showed that compared with contralateral healthy eyes, changes in corneal endothelial cell density and average area near fresh KP were statistically significant [12]. In this study, no significant difference in corneal endothelial cell density and average area was found between eyes with KP and eyes without KP. Due to the statistical significance of the interaction between the course of disease, anterior chamber inflammatory cells and KP, combined with the corneal endothelial cell density mean diagram, with or without anterior chamber inflammatory cells or KP, the corneal endothelial cell density was higher in the eyes with or without anterior chamber inflammatory cells or KP than in the eyes with or without anterior chamber inflammatory cells or KP, while other factors did not have such definite effects, which was consistent with the statistical analysis of the main effects of various factors on corneal endothelial cell density. Therefore, we think that in the interaction of the three factors, the role of the course of disease is more prominent, but it will be affected and restricted by the other two factors.

Few studies have been conducted on the factors affecting corneal endothelial cell parameters in uveitis, and a longitudinal study on a sufficient sample of patients with uveitis is needed to further study these factors.

### 4.4. Correlation of Corneal Endothelial Cell Parameters

Our study confirmed that the area of corneal endothelial cells in children and adolescents with uveitis was positively correlated with the coefficient of variation of cell area. A negative correlation was found between the percentage of hexagonal cells and the coefficient of variation of cell area in the affected eye. The cell area of the affected eye was negatively correlated with the density of corneal endothelial cells. The findings are consistent with clinical tests. Therefore, the measurement of corneal endothelial cell density has the potential to be used as an indicator of corneal endothelial health, which is consistent with our conclusion [16].

### 4.5. Clinical Application of a Corneal Endothelial Microscope in Children with Uveitis

Corneal endothelial microscopy is an objective evaluation technique, which is mainly used in cell counting before cataract surgery, but rarely in uveitis, neglecting its value in this potential application. Corneal endothelial microscopy allows direct observation and analysis of the morphological images of corneal endothelial cells by photography, and combined with the IMAGEnet system can also allow objective quantitative analysis.

To summarize our observation results, using the corneal endothelial microscope to observe and evaluate the corneal endothelial morphology of uveitis is objective, and facilitates observation of abnormality or changes in corneal endothelial cells which cannot be detected by slit lamp examination. Therefore, the morphological images of KP and endothelial cells under the corneal endothelial microscope and the analysis of various parameters have practical clinical significance for diagnosis and accurate judgment of the outcome of the disease.

In general, the surgical treatment of uveitis complications in children is less effective than in adults, and the visual prognosis of patients depends on the control of inflammation and the effective management of complications. Children with cataract should be treated with cataract surgery under complete control of inflammation, then the injury of corneal endothelium before operation will affect the safety of operation and the recovery of visual acuity after the surgery [17]. Corneal endothelial microscopy can also provide objective indicators for the evaluation of inflammation control with applications in intraocular surgery for other complications of uveitis in children.

The noncontact corneal endothelial microscope has limitations. For example, unlike confocal microscopy, it is possible to observe and record the whole corneal layer, corneal thickness, corneal nerve density, corneal light scattering and corneal cell density from epithelial cells to endothelial cells. It can also provide histological information on cell appearance and density. However, the noncontact corneal endothelial microscope can capture images of endothelial cells without contact with the cornea, which avoids the risk of disease transmission in the measurement process, reduces the potential eye damage, and greatly improves patients' comfort of patients, and is inexpensive. In line with the conditions of epidemiological screening methods, it is suitable for general examination of patients with uveitis.

The present study also has some limitations. Firstly, the corneal endothelium in children with uveitis was not prospectively observed in order to understand the dynamic changes of corneal endothelium in different periods of inflammation. Secondly, due to the small number of cases with definite classification and diagnosis, it was not possible to observe and compare the corneal endothelial cell changes in different types of uveitis in children to understand their different characteristics. Finally, because noncontact corneal endothelial microscopy is difficult to obtain images in the cornea with edema, thickening, and corneal scarring, and only provides images of the corneal endothelial layer, in contrast, in vivo confocal microscopy can analyze all corneal layer cells and show the microstructure changes of the cornea. We will use confocal microscopy in vivo to study pediatric uveitis in more detail. Therefore, on this basis, further study is needed to understand the mechanism by which corneal endothelial cells are damaged in uveitis, and ultimately reduce corneal injury and to protect vision in this disease.

In summary, our study demonstrated that corneal endothelial microscopy can observe the abnormal morphology and structure of corneal endothelium in children with uveitis, and can detect small KP which cannot be detected by slit lamp examination. In addition, the significant effect of uveitis on the morphological parameters of corneal endothelial cells in children indicates that each clinical index can assist in the diagnosis of uveitis in children. Finally, we showed that the qualitative and quantitative analysis of corneal endothelial cells using corneal endothelial microscopy and the IMAGEnet system has practical clinical significance for the diagnosis, prognosis and preoperative evaluation of complications in children with uveitis.

## Figures and Tables

**Table 1:** General information on eye grouping of children with uveitis.

Group	Affected eyes	Average age	Best corrected visual acuity	Disease course
A_1_	35	10.17 ± 3.52	HM/20 cm~1.2	1 month~1 year
A_2_	77	12.49 ± 4.22	HM/20 cm~1.5	14 months~15 years
B_1_	64	12.08 ± 4.65	HM/20 cm~1.5	1.5 months~15 years
B_2_	48	11.35 ± 3.34	CF/10 cm~1.2	1 month~9 years
C_1_	69	11.75 ± 4.72	HM/20 cm~1.5	1 month~15 years
C_2_	43	11.79 ± 3.04	HM/20 cm~1.2	1 month~9 years

A_1_: short disease course group; A_2_: long disease course group; B_1_: group without inflammatory cells in the anterior chamber; B_2_: group with inflammatory cells in the anterior chamber; C_1_: non-KP group; C_2_: KP group.

**Table 2:** Comparison of corneal endothelial cell parameters between the uveitis group and the control group.

Corneal endothelial cell parameters	Uveitis group (*n* = 28)	Control group (*n* = 28)	*t*	*P* values
CD	2843.1 ± 465.7	3182.8 ± 253.7	-3.966	<0.001
AVE	367.6 ± 105.9	316.2 ± 26.0	2.657	0.013
CV	32.1 ± 6.4	28.6 ± 5.2	2.097	0.046
PHC	59.8 ± 14.5	66.9 ± 12.5	-2.516	0.018

Notes: independent sample *t*-test. *P* < 0.05 denoted statistical significance. Data are shown as mean ± standard deviation. CD: cell density; AVE: average cell area; CV: coefficient of variation of the average cell area; PHC: percentage of hexagonal cells.

**Table 3:** Comparison of corneal endothelial cell parameters between male and female children with uveitis.

Corneal endothelial cell parameters	Male (*n* = 46)	Female (*n* = 66)	*t*	*P* values
CD	2993.3 ± 314.9	2994.5 ± 380.7	-0.018	0.985
AVE	337.8 ± 36.8	342.4 ± 73.9	-0.392	0.696
CV	30.6 ± 8.7	31.1 ± 6.8	-0.368	0.714
PHC	62.6 ± 10.0	61.6 ± 12.9	0.466	0.642

Notes: independent sample *t*−test. *P* < 0.05 denoted statistical significance. Data are shown as mean ± standard deviation. CD: cell density; AVE: average cell area; CV: coefficient of variation of the average cell area; PHC: percentage of hexagonal cells.

**Table 4:** Comparison of corneal endothelial cell parameters between the left and right eyes with uveitis.

Corneal endothelial cell parameters	Right eye (*n* = 56)	Left eye (*n* = 56)	*t*	*P* values
CD	3021.7 ± 259.7	2966.3 ± 428.3	0.828	0.409
AVE	333.3 ± 28.7	347.8 ± 81.6	-1.248	0.216
CV	30.3 ± 6.6	31.4 ± 8.5	-0.765	0.446
PHC	62.7 ± 10.6	61.3 ± 12.8	0.602	0.549

Notes: independent sample *t*-test. *P* < 0.05 denoted statistical significance. Data are shown as mean ± standard deviation. CD: cell density; AVE: average cell area; CV: coefficient of variation of the average cell area; PHC: percentage of hexagonal cells.

**Table 5:** Observation of corneal endothelial cell parameters in children with uveitis at different levels.

Group	Short-course group (A_1_)		Long-course group (A_2_)	
	No inflammatory cells in the anterior chamber (B_1_)	Inflammatory cells in the anterior chamber (B_2_)	No inflammatory cells in the anterior chamber (B_1_)	Inflammatory cells in the anterior chamber (B_2_)
Non-KP group (C_1_) CD (PCS/mm^2^)	3010.3 ± 270.7	3171.9 ± 354.9	2937.5 ± 380.0	2918.2 ± 362.5
KP group (C_2_) CD (PCS/mm^2^)	3689.5 ± 12.8	3030.3 ± 413.8	2932.5 ± 393.6	3010.5 ± 215.8
Non-KP group (C_1_) AVE (*μ*m^2^)	334.6 ± 29.7	318.4 ± 33.4	350.2 ± 84.0	348.0 ± 48.6
KP group (C_2_) AVE (*μ*m^2^)	271.1 ± 0.9	338.0 ± 64.5	346.7 ± 48.3	333.8 ± 24.0
Non-KP group (C_1_) CV (%)	28.7 ± 3.2	30.9 ± 9.0	30.7 ± 4.7	28.2 ± 7.5
KP group (C_2_) CV (%)	39.1 ± 4.9	33.6 ± 9.6	33.2 ± 5.0	30.3 ± 12.3
Non-KP group (C_1_) PHC (%)	69.1 ± 8.2	63.0 ± 10.5	63.3 ± 11.6	61.4 ± 7.4
KP group (C_2_) PHC (%)	59.0 ± 2.8	55.5 ± 15.7	57.6 ± 7.9	61.2 ± 12.9

Notes: data are shown as mean ± standard deviation. CD: cell density; AVE: average cell area; CV: coefficient of variation of the average cell area; PHC: percentage of hexagonal cells.

**Table 6:** Analysis results of the influence of various factors on corneal endothelial cell parameters of children with uveitis.

Factor	A	B	C	A × B	A × C	B × C	A × B × C
*F*_CD_	8.987	1.422	2.883	2.285	1.497	3.862	6.224
*F*_AVE_	3.146	0.294	0.882	1.002	0.159	1.219	2.042
*F*_CV_	1.505	1.146	4.842	0.076	1.094	1.000	0.320
*F*_PHC_	0.058	0.415	3.695	0.847	0.902	0.434	0.056
*P*_CD_	0.003	0.236	0.092	0.134	0.224	0.052	0.014
*P*_AVE_	0.079	0.589	0.350	0.319	0.691	0.272	0.156
*P*_CV_	0.223	0.287	0.030	0.783	0.298	0.320	0.368
*P*_PHC_	0.081	0.521	0.057	0.359	0.344	0.512	0.813

A: disease course; B: inflammatory cells in the anterior chamber; C: KP. CD: cell density; AVE: average cell area; CV: coefficient of variation of the average cell area; PHC: percentage of hexagonal cells.

## Data Availability

The datasets used and/or analyzed during the present study are available from the corresponding author on reasonable request.

## References

[1] Zierhut M., Michels H., St??biger N., Besch D., Deuter C., Heiligenhaus A. (2005). Uveitis in children. *International Ophthalmology Clinics*.

[2] Tugal-Tutkun I. (2011). Pediatric uveitis. *Journal of Ophthalmic and Vision Research*.

[3] Kalinina Ayuso V., Scheerlinck L. M., de Boer J. H. (2013). The effect of an Ahmed glaucoma valve implant on corneal endothelial cell density in children with glaucoma secondary to uveitis. *American Journal of Ophthalmology*.

[4] Corneal endothelial photography (1997). Corneal Endothelial Photography: Three-year Revision. *Ophthalmology*.

[5] Hatou S. (2011). Hormonal regulation of Na+/K+-dependent ATPase activity and pump function in corneal endothelial cells. *Cornea*.

[6] Zhu Q., Zhu Y., Tighe S., Liu Y., Hu M. (2019). Engineering of human corneal endothelial CellsIn vitro. *International Journal of Medical Sciences*.

[7] Joyce N. C. (2012). Proliferative capacity of corneal endothelial cells. *Experimental Eye Research*.

[8] Price M. O., Mehta J. S., Jurkunas U. V., Price F. W. (2021). Corneal endothelial dysfunction: evolving understanding and treatment options. *Progress in Retinal and Eye Research*.

[9] Garza-Leon M. (2016). Corneal endothelial cell analysis using two non-contact specular microscopes in healthy subjects. *International Ophthalmology*.

[10] Cheung S. W., Cho P. (2000). Endothelial cells analysis with the TOPCON specular microscope SP-2000P and IMAGEnet system. *Current Eye Research*.

[11] Bartakova A., Alvarez-Delfin K., Weisman A. D. (2016). Novel identity and functional markers for human corneal endothelial cells. *Investigative Ophthalmology & Visual Science*.

[12] Pillai C. T., Dua H. S., Azuara-Blanco A., Sarhan A. R. (2000). Evaluation of corneal endothelium and keratic precipitates by specular microscopy in anterior uveitis. *The British Journal of Ophthalmology*.

[13] LaMattina K. C., Goldstein D. A. (2017). Adalimumab for the treatment of uveitis. *Expert Review of Clinical Immunology*.

[14] Commodaro A. G., Bombardieri C. R., Peron J. P. (2010). p38*α* MAP kinase controls IL-17 synthesis in Vogt-Koyanagi-Harada syndrome and experimental autoimmune uveitis. *Investigative Ophthalmology & Visual Science*.

[15] Shivanna M., Srinivas S. P. (2009). Microtubule stabilization opposes the (TNF-*α*)-induced loss in the barrier integrity of corneal endothelium. *Experimental Eye Research*.

[16] Tekin K., Sekeroglu M. A., Kiziltoprak H., Yilmazbas P. (2017). Corneal densitometry in healthy corneas and its correlation with endothelial morphometry. *Cornea*.

[17] Yangzes S., Seth N. G., Singh R. (2019). Long-term outcomes of cataract surgery in children with uveitis. *Indian Journal of Ophthalmology*.

